# Large spin Hall angle in vanadium film

**DOI:** 10.1038/s41598-017-01112-9

**Published:** 2017-05-02

**Authors:** T. Wang, W. Wang, Y. Xie, M. A. Warsi, J. Wu, Y. Chen, V. O. Lorenz, X. Fan, J. Q. Xiao

**Affiliations:** 1 Department of Physics and Astronomy, University of Delaware, Newark, Delaware 19716 USA; 2 Department of Physics, University of Illinois at Urbana-Champaign, Urbana, Illinois 61801 USA; 3 Department of Physics and Astronomy, University of Denver, Denver, Colorado 80208 USA

## Abstract

We report a large spin Hall angle observed in vanadium films sputter-grown at room temperature, which have small grain size and consist of a mixture of body centered tetragonal (bct) and body centered cubic (bcc) structures. The spin Hall angle is as large as *θ*_*V*_ = −0.071 ± 0.003, comparable to that of platinum, *θ*_*Pt*_ = 0.076 ± 0.007, and is much larger than that of bcc V film grown at 400 °C, *θ*_*V*_*bcc*_ = −0.012 ± 0.002. Similar to *β*-tantalum and *β*-tungsten, the sputter-grown V films also have a high resistivity of more than 200 *μ*Ω∙cm. Surprisingly, the spin diffusion length is still long at 16.3 nm. This finding not only indicates that specific crystalline structure can lead to a large spin Hall effect but also suggests 3*d* light metals should not be ruled out in the search for materials with large spin Hall angle.

## Introduction

The efficient generation of pure spin current is a key enabling technology to develop future memory and logic devices with low energy consumption^1, 2^. As one viable technique to generate and detect pure spin current, the spin Hall effect (SHE)^3, 4^ has been intensively investigated in the study of spin-orbit interaction in normal metal/ferromagnetic metal (NM/FM) systems. The pure spin current generated from the SHE can be described by ${J}_{S}={\theta }_{SH}^{0}(\hat{\sigma }\times {J}_{C})$, where the material-specific spin Hall angle ${\theta }_{SH}^{0}$ characterizes the spin current conversion efficiency from the charge current *J*_*C*_, and $\hat{\sigma }$ is the spin polarization vector of the pure spin current. One common method to quantify ${\theta }_{SH}^{0}$ in NMs is to employ NM/FM bilayers and to measure the current-driven spin-orbit torques on the FM^5, 6^. In this letter, we use a phenomenological parameter *θ*_*SH*_ to represent the effective spin Hall angle which is extracted from the measured spin-orbit torques in NM/FM bilayers.

To date, most studies have focused on the 4*d* and 5*d* transition metals, since the spin-orbit coupling strength of individual atoms scales as *Z* ^4, 7, 8^ where *Z* is the atomic number. Large spin Hall angles have been observed in heavy metals such as Pt^9, 10^, *β*-Ta^11^, *β*-W^12, 13^, Hf^14, 15^, etc. Considerable efforts have also been focused on enhancing the conversion efficiency by introducing external scattering mechanisms in the heavy metals, which has lead to the observation of giant spin Hall angles in CuBi alloys^16^, AuW^17^, CuIr^18^, CuPd^19^, etc. Due to their relatively low *Z*, 3*d* light transition metals are often neglected in the search for efficient spin Hall materials. However, very recently, Du *et al*. observed significant spin pumping-driven inverse SHE (ISHE) voltages in YIG/Cr bilayers, and obtained a spin Hall angle as large as −0.051 ± 0.005^20^. Qu *et al*. have also demonstrated sizeable ISHE in Cr by using a thermal spin injection method^21^. In this letter, the spin-orbit torques (SOTs) in V films has been characterized by using an optical spin torque magnetometer based on the polar magneto-optical Kerr effect (MOKE)^6, 22^. A large spin Hall angle of −0.071 ± 0.003 has been found in V/Co_40_Fe_40_B_20_ bilayers. As comparison, the spin Hall angles find in Ta/Co_40_Fe_40_B_20_ and Pt/Co_40_Fe_40_B_20_ by using the same MOKE setup are −0.139 ± 0.003 and 0.076 ± 0.007, respectively. The large spin Hall angle appears to correlate to the structure of the V layer, which consists of body centered tetragonal (bct) and body centered cubic (bcc) phases. Unlike *β*-Ta and *β*-W films, these room-temperature sputter-grown V films still have a long spin diffusion length of 16.3 nm. Vanadium films grown at high temperature exhibit dominant bcc structure and a much smaller spin Hall angle of *θ*_*V*___*bcc*_ = −0.012 ± 0.002, which is comparable to the reported value of *θ*_*V*_ = −0.010 ± 0.001^20^.

## Results

The V/CoFeB bilayer films used in this study were deposited by direct current (DC) magnetron sputtering on thermally oxidized silicon substrates at room temperature with a base pressure of less than 3 × 10^−7^ Torr. The nominal composition of CoFeB is Co:Fe:B = 40:40:20. A capping layer of SiO_2_ was deposited by radio frequency (RF) magnetron sputtering. Five samples were prepared with different V thicknesses: samples A–E as ||V(x)/CoFeB(2)/SiO_2_(5) with x = 2, 5, 10, 30, 50 nm (“||” denotes the substrate end, and the values in parentheses represent the thicknesses in nm). The deposition rates and sputtering power were 0.067 nm/s and 18 W for CoFeB and 0.070 nm/s and 24 W for V, respectively. The pressure was maintained at 3.0 mTorr. One control sample F || V(30)/CoFeB(2)/SiO_2_(5) was fabricated at 400 °C with a lower base pressure of 8 × 10^−8^ Torr.

**Figure 1:**
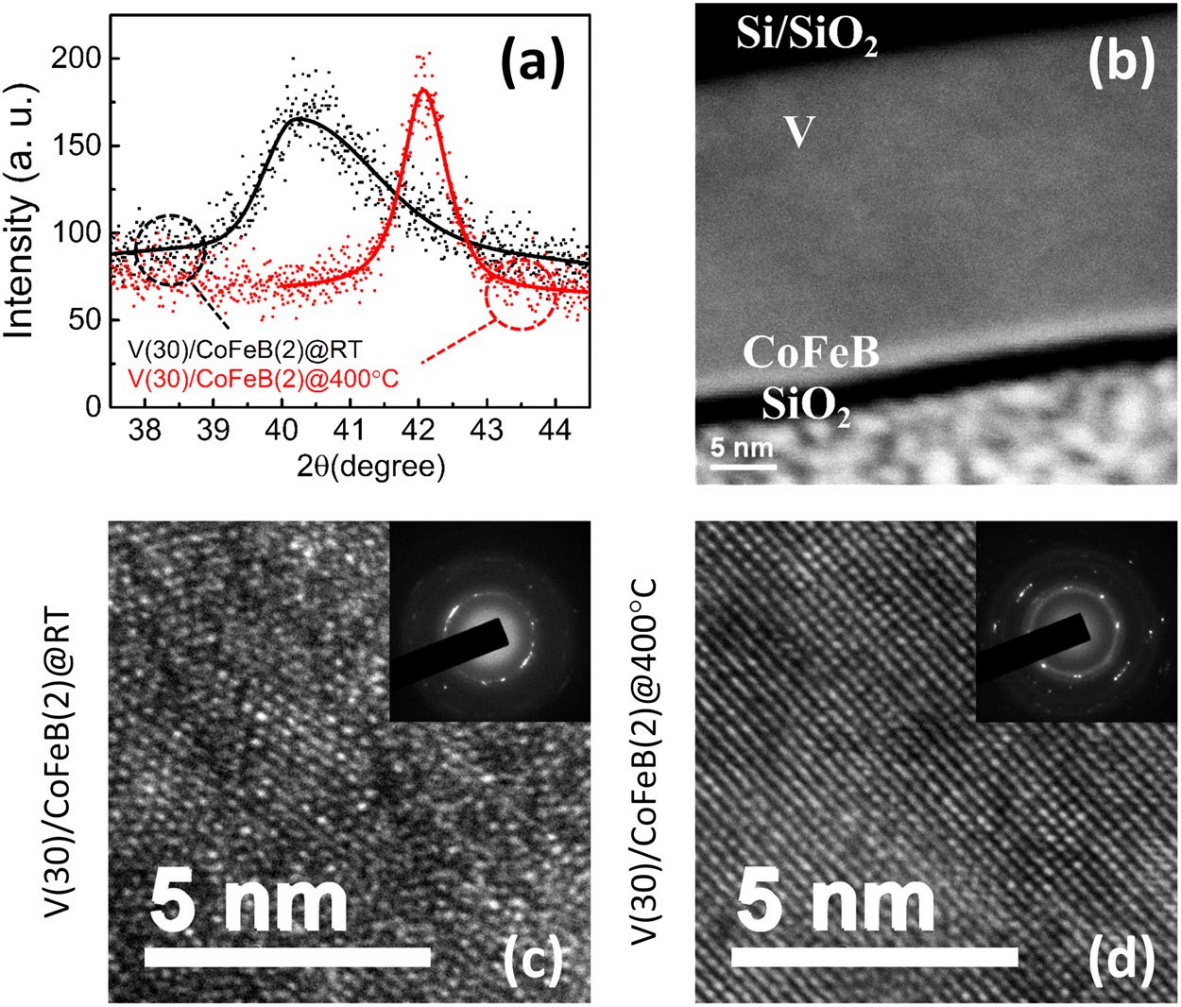
(**a**) X-ray diffraction patterns for sample D, V(30)/CoFeB(2) grown at room temperature (black) and sample F, V(30)/CoFeB(2) grown at 400 °C (red). (**b**) Cross section STEM image of sample D. (**c**) and (**d**) TEM images of samples D and F, respectively. The insets are the corresponding electron diffraction (ED) patterns.

Figure 1(a) shows X-ray diffraction (XRD) patterns of samples D and F, which have the same 30 nm V thickness, but were grown at room temperature and 400 °C, respectively. Sample D shows a broad and asymmetric diffraction peak with the center located at 40.3° whereas the main diffraction peak of sample F is at 42.1°. Figure 1(b) shows the scanning transmission electron microscopy (STEM) cross section view of sample D. Figure 1(c) and (d) show the transmission electron microscopy (TEM) and electron diffraction (ED) patterns of samples D and F, respectively. In sample D, the average grain size is about 5 nm and the interlayer spacing varies from 2.20 Å to 2.31 Å at different locations. Sample F has a larger grain size above 10 nm, and the interlayer spacing is dominantly 2.16 Å.

**Figure 2:**
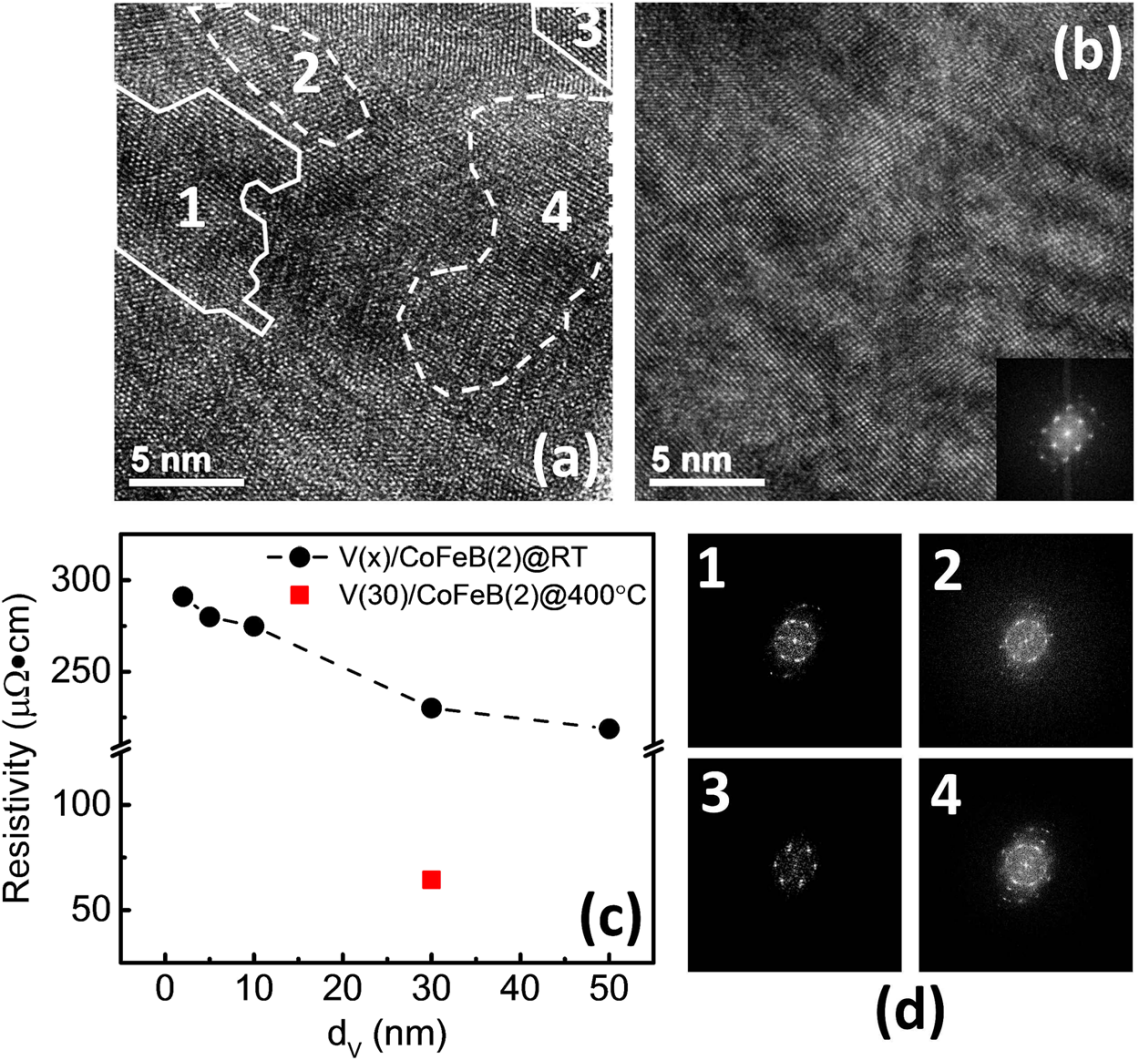
(**a**) HRTEM micrographs of sample D, showing both bct and bcc phases, which are marked by white solid lines and dashed lines, respectively. (**b**) HRTEM micrographs of sample F, prepared at 400 °C; the inset shows a clear bcc FFT pattern. (**c**) The total resistivity of test samples A-E (black) and control sample F (red) as a function of the V thickness. (**d**) The FFT images of regions 1–4 in (**a**). (d1) and (d3) could be best indexed as having bct structure, while (d2) and (d4) show a bcc V structure.

To better characterize the V structure in our samples, we performed fast Fourier transform (FFT) analysis based on high resolution transmission electron microscopy (HRTEM) images in Fig. 2(a) and (b). The structure from the grains surrounded by white solid curves in Fig. 2(a) can be best indexed by a [111] zone axis of a bct V^23, 24^, whereas the grains surrounded by dashed curves can be best described by a bcc V. These analyses suggest the sputter-grown V films at room temperature are a mixture of bct and bcc structures, which may also explain the broad XRD peak in Fig. 1(a). This is similar to *β*-Ta films, which have tetragonal nanocrystalline phase in an amorphous matrix^25^, while *α*-Ta films have bcc structure. In sharp contrast, as shown in Fig. 2(b), sample F grown at 400 °C shows dominant bcc V structure from the FFT analyses. As shown in Fig. 2(c), the resistivities of samples A - E, all grown at room temperature, vary from 290 *μ*Ω∙cm to 220 *μ*Ω∙cm as the V thickness changes from 2 to 50 nm. On the other hand, sample F, grown at 400 °C, shows much reduced resistivity.

**Figure 3:**
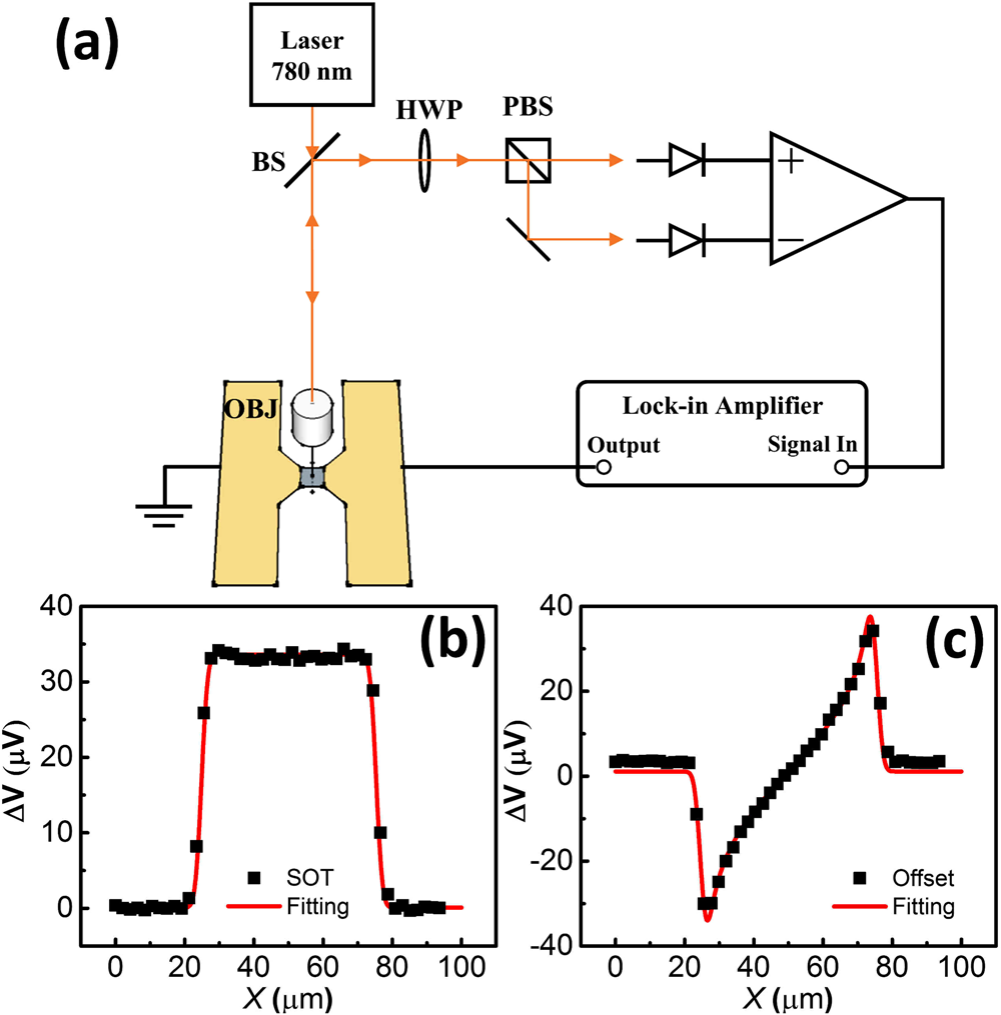
(**a**) Experimental setup for the SOT measurement. (BS, beamsplitter; HWP, half-wave plate; OBJ, objective; PBS, polarizing beamsplitter). The Kerr rotation angle between the incident and reflected laser is detected by the balance detector. (**b**) The SOT signal and (**c**) Oersted field generated from the current across the sample width. The sample is located between 25 µm and 75 µm.

Polar MOKE measurements of current-driven spin-orbit torque in V(x)/CoFeB(2)/SiO_2_(5) samples were performed using the experiment setup shown in Fig. 3(a). The bilayer was patterned into a 50 *μ*m × 50 *μ*m strip. An AC current was sent through the sample. The current in the V layer generated an out-of-plane Oersted field and an effective field due to spin-orbit torque, which cause a change of the magnetization Δ*m*_z_ in the CoFeB layer. The change of the magnetization was detected by measuring the polarization change in a laser beam with 2 *µ*m diameter. The MOKE voltage signal consists of SOT (Fig. 3(b)) and out-of-plane Oersted field terms (Fig. 3(c)) which can be separately extracted based on the symmetry with respect to the external magnetic field^6, 22^.

**Figure 4:**
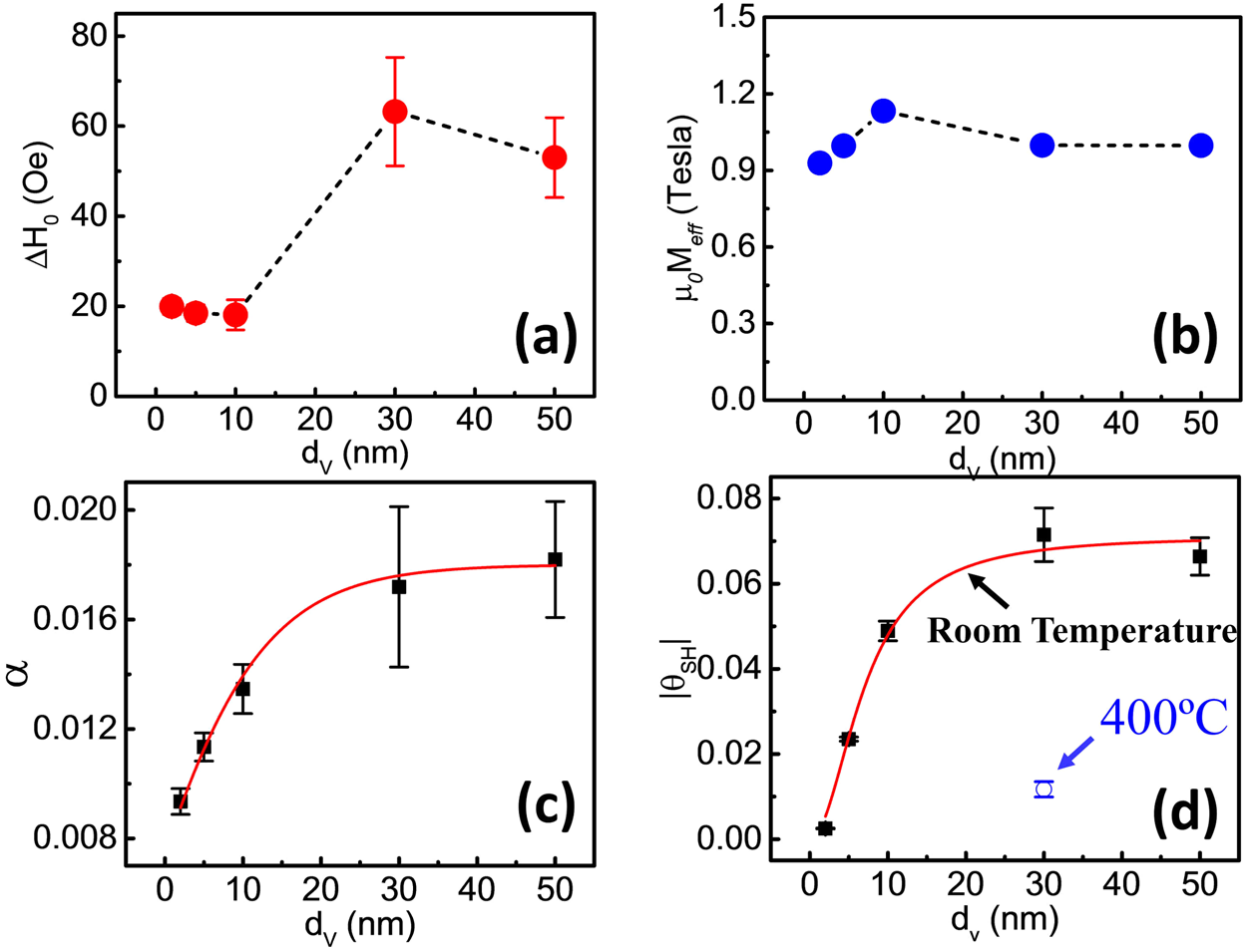
The FMR and MOKE measurement results of samples A-E. The V thickness dependence of (**a**) inhomogeneous broadening Δ*H*_0_ (which indicates film quality and inhomogeneity), (**b**) the effective magnetization, (**c**) the damping constant, (**d**) the spin Hall angles of samples A-E, and the control sample F (marked with the open blue circle).

In order to extract the spin diffusion length *λ*_*sf*_ of the V layer, we analyzed the dependence of the Gilbert damping coefficient *α* of the CoFeB layer as a function of the V layer thickness using a spin pumping experiment^26–28^. The inhomogeneous broadening (Δ*H*_0_) and the effective magnetization (*μ*_0_ *M*_*eff*_) are shown in Fig. 4(a) and (b), respectively. Δ*H*_0_ (defined as the zero-frequency intercept of the FMR linewidth) indicates the V film quality and inhomogeneity. The five V films exhibit film quality fluctuations. The effective magnetization field ${\mu }_{0}{M}_{eff}={\mu }_{0}{M}_{S}-\frac{2{K}_{\perp }}{{\mu }_{0}{M}_{S}}$ is related to the perpendicular anisotropy field, which may vary for different interfacial conditions, *μ*_0_ is the permeability of vacuum, *M*_*S*_ is the saturation magnetization, and *K*_⊥_ is the surface anisotropy energy density. The damping constant *α* as a function of the V layer thickness *d*_*V*_ is plotted in Fig. 4(c). The damping constant increases with the V layer thickness, and saturates above 30 nm of V. The increase of the damping constant due to the V layer $\alpha ^{\prime} ({d}_{V})=\alpha ({d}_{V})-\alpha ({d}_{V}=0)$ can be described as^29, 30^:

1 $$\frac{{\alpha }^{^{\prime} }({d}_{V})}{{\alpha }^{^{\prime} }({d}_{V}={\rm{\infty }})}=\frac{1+{\varepsilon }^{-1/2}}{1+{\varepsilon }^{-1/2}\tanh {({d}_{V}/{\lambda }_{sf})}^{-1}}$$

where *ε* = *τ*_*el*_/*τ*_*sf*_ is the spin flip probability for each scattering event, *τ*_*el*_ is the elastic scattering time, and *τ*_*sf*_^−1^ is the spin-flip rate. The red curve in Fig. 4(c) is the fitting curve, and the extracted spin diffusion length *λ*_*sf*_  = 16.3 ± 0.7 nm, which is comparable to the published value of *λ*_*sf*_  = 14.9 ± 2.4 nm^20^.

The spin Hall angle *θ*_*m*_ can be extracted from the damping like spin Hall torque measured by MOKE magnetometer, using ${\theta }_{m}({d}_{V})={J}_{S}/{J}_{C}=(\frac{{h}_{SOT}}{{I}_{C}/w})(\frac{2e}{\hslash }){\mu }_{0}{M}_{S}{d}_{CoFeB}{d}_{V}$ ^6^, where *h*_*SOT*_ is the out-of-plane effective field, an electrical current *I*_*C*_ flows through samples with the sample width *w* = 50 *μ*m, and *d*_*CoFeB*_ is 2 nm in the MOKE measurement. Due to the complexity of the current distribution in the bilayer structure and the large resistivity of the 2 nm CoFeB layer, here we make a simplifying assumption that all the charge current *I*_*C*_ flows through the V, which underestimates the spin Hall angle but specifies a lower bound. The saturation magnetization *μ*_0_ *M*_*S*_ = 1.60 *T*, extracted from another 40 nm CoFeB sample through a FMR measurement. As shown in Fig. 4(d), the spin Hall angle increases with the V layer thickness and approaches saturation as the V thickness goes above the V spin diffusion length. In order to account for spin transparency and interface coupling, we use the modified spin transport model to extract the spin Hall angle *θ*_SH_(∞)^30^, the V thickness dependence of the measured spin Hall angle *θ*_*m*_(*d*_*V*_) becomes:

2 $$\frac{{\theta }_{m}({d}_{V})}{{\theta }_{SH}({\rm{\infty }})}=\frac{\cosh ({d}_{V}/{\lambda }_{sf})-1}{\cosh ({d}_{V}/{\lambda }_{sf})+R}$$

where $R=\frac{{G}_{V}}{2{G}_{\uparrow \downarrow }}\sinh (\frac{{d}_{V}}{{\lambda }_{sf}})$ ^31^. The extracted spin Hall angle is *θ*_*SH*_(∞) = −0.071 ± 0.003, with the fitting parameter *R* = −0.908 ± 0.017. On the other hand, the control sample F, with its V layer grown at 400 °C, has a measured spin Hall angle of *θ*_*m*_(*d*_*V* _= 30 nm) = −0.012 ± 0.002, which is comparable to the reported value of V film^20^. The non-zero *R* indicates the complex interfacial condition at the V/CoFeB interface, which could be caused by spin backflow (SBF) and/or enhanced spin scattering^32–35^.

## Discussion

It has been found that the spin transparency at the NM/FM interface can play a critical role in determining the spin torque efficiency^32–35^. The insertion of atomically thin magnetic layers at a Pt/Py interface^32^, or one ultra-thin Hf layer between Pt/CoFeB could significantly modulate the interfacial transparency and enhance the spin injection efficiency from Pt to the FM layer^33^. Due to the importance of the interfacial condition, we have analyzed the spin mixing conductance of the V/CoFeB interface. The effective spin mixing conductance is ${g}_{eff}^{\uparrow \downarrow }=\frac{4\pi {M}_{S}{d}_{CoFeB}}{\gamma \hslash }(\alpha -{\alpha }_{0})$, and ${g}_{eff}^{\uparrow \downarrow }=\frac{h}{{e}^{2}}{G}_{eff}^{\uparrow \downarrow }$ ^34^, where *e* is the elementary charge, *γ* is the gyromagnetic ratio, *h* and *ħ* are Planck and reduced Planck constants, respectively. The bare spin mixing conductance ${G}^{\uparrow \downarrow }=\frac{{G}_{eff}^{\uparrow \downarrow }}{1-2{G}_{eff}^{\uparrow \downarrow }/{G}_{V}}$, where ${G}_{V}={({\rho }_{V}{\lambda }_{sf})}^{-1}$ ^34^, *ρ*_*V*_ represents the resistivity. We obtain ${G}_{eff}^{\uparrow \downarrow }=(0.25-1.45)\times {10}^{15}\,{{\rm{\Omega }}}^{-1}\,{m}^{-2}$ from spin pumping measurements for samples grown at room temperature and the conductance of the V layer ${G}_{V}=(2.04-3.06)\times {10}^{13}\,{{\rm{\Omega }}}^{-1}\,{m}^{-2}$. The value of ${G}_{eff}^{\uparrow \downarrow }$ is two orders of magnitudes larger than *G*_*V*_, making the bare spin mixing conductance ${G}^{\uparrow \downarrow }$ < 0. This unphysical negative value indicates that there may be other additional magnetic damping enhancement mechanisms at the V/CoFeB interface, which could lead to the overestimation of ${G}_{eff}^{\uparrow \downarrow }$ ^34^. Due to the complication at the V/CoFeB interface, it becomes difficult to extract the spin Hall angle of V. However, under the assumption of a completely transparent interface $\frac{{\theta }_{m}({d}_{V})}{{\theta }_{SH}({\rm{\infty }})}=\frac{\cosh ({d}_{V}/{\lambda }_{sf})-1}{\cosh({d}_{V}/{\lambda }_{sf})}$ ^10^, it is still reasonable to quantify a lower bound of the effective spin Hall angle as *θ*_*V*_ = −0.069 ± 0.002. Because of the transparent interface assumption, the fitting spin diffusion length *λ* = 5.2 ± 0.3 *nm* doesn’t match with *λ*_*sf*_ = 16.3 ± 0.7 nm, which has been extracted from spin pumping experiment by taking account of a non-transparent interface condition.

Previous research has related a large spin Hall angle with specific crystal structures^11, 12, 36^. For example, a giant spin Hall angle *θ*_*SH*_ = −0.12 ~ −0.15 has been reported in *β*-Ta^11^, which has a stretched tetragonal crystal structure with an enlarged lattice constant and a higher resistivity of 190 *µ*Ω·cm compared with *α*-Ta. Similar behavior has also been observed in *β*-W^12^. As a group 5 element, V has a similar Fermi surface as those of Nb and Ta^37^. We therefore speculate the mechanism for the large spin Hall angle in V films is also due to the presence of a tetragonal phase, similar to *β*-Ta^25^. However, unlike *β*-Ta and *β*-W, these sputter-grown V films still have a long spin diffusion length.

In summary, a large spin Hall angle is observed in 3*d* light transition metal V, which is deposited at room temperature and characterized with small grain size and enlarged interlayer spacing with mixed bct and bcc states. The spin Hall angle is at least *θ*_*V*_ = −0.071 ± 0.003, comparable to that of Pt, and is much larger than that in bcc V film grown at 400 °C. Similar to *β*-Ta and *β*-W, the V films with mixed bct and bcc phases also show high resistivity. However, the spin diffusion length is still as long as 16.3 nm. The surprisingly large spin Hall angle in V will not only be useful for potential applications in spin-orbit-torque-based magnetization switching, but also have ramifications on understanding the origin of the spin Hall angle. In particular, this research suggests that light metals should not be ruled out in the search for efficient spin Hall materials with large spin Hall angle.
